# Case Report: A Case of Encephalopathy Presenting the Lentiform Fork Sign on MRI in a Diabetic Dialysis Patient

**DOI:** 10.12688/f1000research.25597.2

**Published:** 2021-10-20

**Authors:** Yuri Ishizaki, Ryuzoh Nishizono, Masao Kikuchi, Hiroko Inagaki, Yuji Sato, Shouichi Fujimoto

**Affiliations:** 1 Department of Nephrology, University of Miyazaki Hospital, Faculty of Medicine, University of Miyazaki, 5200 Kihara, Kiyotake, Miyazaki, 889-1692, Japan; 2 Dialysis Division, University of Miyazaki Hospital, Faculty of Medicine, University of Miyazaki, Miyazaki, Japan; 3 Department of Hemovascular Medicine and Artificial Organs, Faculty of Medicine, University of Miyazaki, Miyazaki, Japan

**Keywords:** lentiform fork sign, basal ganglia lesion, diabetic uremic syndrome, metformin, consciousness disturbance

## Abstract

Basal ganglia lesions showing an expansile high signal intensity on T2-weighted MRI are termed the lentiform fork sign. This specific finding is mainly observed in diabetic patients with uremic encephalopathy with metabolic acidosis, although there are also reports in patients with ketoacidosis, dialysis disequilibrium syndrome, intoxication, and following drug treatment (e.g., metformin). A 57-year-old Japanese man on chronic hemodialysis for 4 years because of diabetic nephropathy was admitted to our hospital for relatively rapid-onset gait disturbance, severe dysarthria, and consciousness disturbance. Brain T2-weighted MRI showed the lentiform fork sign. Hemodialysis was performed the day before admission, and laboratory tests showed mild metabolic (lactic) acidosis, but no uremia. Surprisingly, metformin, which is contraindicated for patients with end-stage kidney disease, had been prescribed for 6 months in his medication record, and his sluggish speaking and dysarthria appeared gradually after metformin treatment was started. Thus, the encephalopathy was considered to be related to metformin treatment. He received hemodialysis treatment for 6 consecutive days, and his consciousness disturbance and dysarthria improved in 1 week. At the 8-month follow-up, the size of the hyperintensity area on MRI had decreased, while the mild gait disturbance remained. Considering the rapid onset of gait and consciousness disturbance immediately before admission, diabetic uremic syndrome may also have occurred with metformin-related encephalopathy, and resulted in the lentiform fork sign, despite the patient showing no evidence of severe uremia on laboratory data.

## Introduction

Metabolic encephalopathy with abnormal basal ganglia lesions has been reported in hemodialysis patients. Ingestion of some types of mushroom, star fruit, and drugs (e.g., anti-herpes virus drugs) can cause encephalopathy in these patients ^1– 3^. In particular, diabetic dialyzed patients can present with bilateral symmetrical low densities in the basal ganglia on brain computed tomography (CT), with a bilateral symmetrical hyperintensity in the same area and a lentiform fork sign on T2-weighted MRI ^4– 10^. In addition to diabetic uremic syndrome (DUS) ^4, 5^, the lentiform fork sign can be observed in severe metabolic acidosis ^11– 13^, dialysis disequilibrium syndrome ^14^, and metformin-associated encephalopathy (ME) ^6, 7^. The pathogenic basis of this sign is considered to relate to cytotoxic edema based on the severity of metabolic acidosis ^8, 11^. Intensive dialysis is a therapeutic option for removing the uremic toxins, to correct metabolic acidosis and remove medications. Herein, we present a case of a 57-year-old Japanese man in whom the lentiform fork sign was a clue for the differential diagnosis of ME or DUS. Metformin tends to increase lactate production and result in metabolic acidosis in ME ^6, 7, 9, 10^, while chronic hyperglycemia with coexistence of uremic toxins and metabolic acidosis is the main mechanism in DUS ^4, 5^. Which of these is the main cause in our case presenting with the lentiform fork sign is discussed below.

## Case report

**Figure 1.**
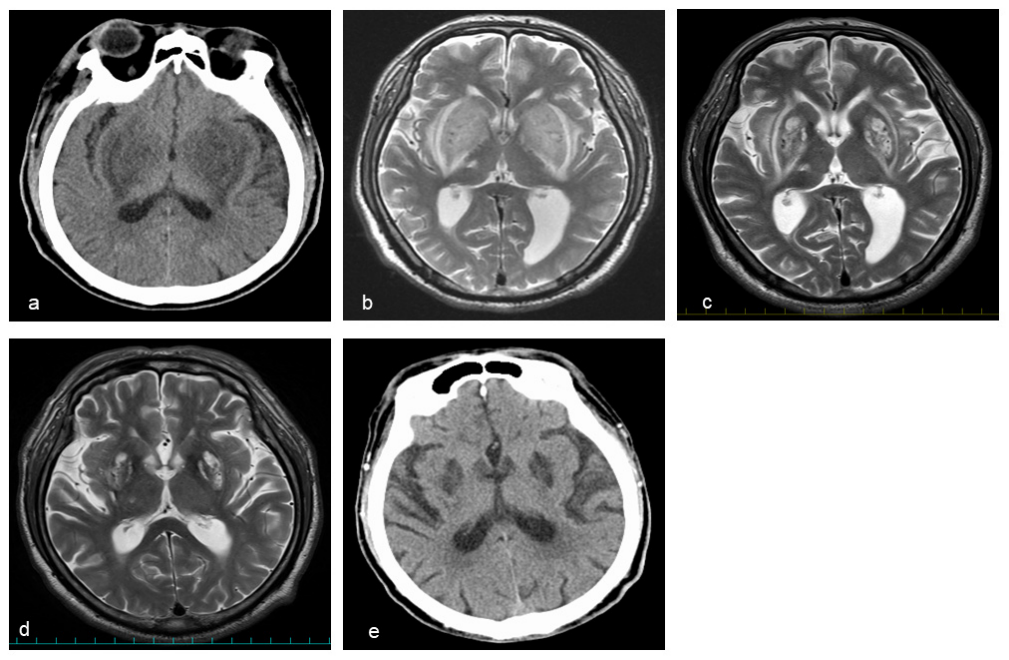
**a**, **e** Head computed tomography (CT) and **b–d** head MRI (T2-weighted image). **a, b** High-resolution lesions in the bilateral symmetrical basal ganglia were evident at admission. **c, d** The bilateral symmetrical basal ganglia lesions gradually improved on the 18 ^th^ hospital day and at three-month follow-up. **e** However, the basal ganglia lesions remained at eight-month follow-up.

A 57-year-old Japanese man who had been on maintenance hemodialysis three-times weekly for four years because of diabetic nephropathy developed gait and consciousness disturbance (the Glasgow Coma Scale score of E3V4M6), fatigue, numbness in his left upper limb, and a slow response during conversation approximately 10 days before admission. His wife denied him taking mushrooms or star fruit, which can cause consciousness disturbance in hemodialysis patients. There were no abnormal neurologic findings on physical examination. However, bilateral symmetrical basal ganglia lesions were noted on brain CT ( Figure 1a).

On admission to our hospital, his consciousness was disturbed, such as he only could open his eyes following calling, and he had difficulty sitting alone. He showed a tonic planter reflex on physical examination. His blood pressure was 190/91 mmHg, and his heart rate was 104 beats per min. Arterial blood gas analysis showed a pH of 7.37, bicarbonate ion of 18.1 mEq/L, and lactic acid of 6.2 mmol/L (normal, 0.5–1.6 mmol/L). Serum vitamin B1 (thiamin) level was 45 ng/mL (normal, 24–66 ng/mL). Serum vitamin B1 (thiamin) level was 45 ng/mL (normal, 24–66 ng/mL). Serum calcium and blood aluminum levels were all within the acceptable range. Kidney function data sampled the day after dialysis, blood urea nitrogen, and serum creatinine were consistent with dialysis. His HbA1c was 5.8% on admission.

Brain MRI showed bilateral symmetrical basal ganglia lesions with an expansile high signal intensity (lentiform fork sign) on T2-weighted sequences ( Figure 1b), which was not seen on MRI taken one-year prior when he developed a right thalamic lacunar infarction.

**Figure 2.**
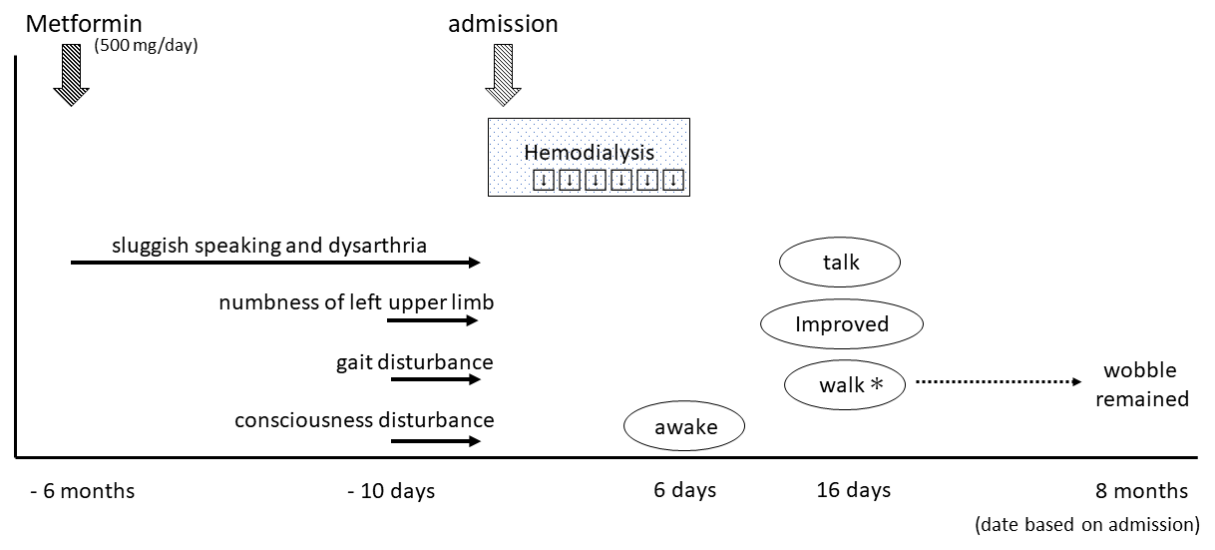
The patient started metformin treatment at six months prior to admission. Since that time, he developed gradual symptoms of sluggish speaking and dysarthria, while numbness of his left upper limb, gait disturbance, and consciousness disturbance appeared 10 days before admission. He received emergency consecutive hemodialysis for six days, after which he awoke, and was gradually able to walk and talk. *Walking was possible, but wobbling during walking remained at eight-month follow-up.

In his medication history, he had taken metformin for six months. His wife said that his sluggish speaking and dysarthria appeared gradually after starting metformin treatment ( Figure 2). His plasma metformin concentration was extremely high (25,700 ng/mL). Thus, we considered that metformin may have initially caused the encephalopathy. However, we also considered the possibility of DUS, because his gait and consciousness disturbance appeared relatively rapidly approximately 10 days before hospitalization. DUS typically occurs in uncontrolled uremic patients with diabetic mellitus.

In either case, we stopped metformin treatment, and immediately performed intensive hemodialysis (four hours per day) for six days after hospitalization to remove metformin and uremic toxin, and to correct metabolic acidosis. The first dialysis session reduced his lactic acid levels from 6.2 to 1.3 mmol/L. After six consecutive sessions of hemodialysis, his consciousness was restored, and his tonic planter reflex disappeared. After starting meals, linagliptin was chosen as an anti-diabetic drug to replace metformin.

On the 18 ^th^ hospital day, T2-weighted brain MRI revealed a modest improvement in the lentiform fork sign ( Figure 1c). The patient was gradually able to sitting alone, walk, and talk with staff and his wife. He was discharged from our hospital within one month.

At three-month follow-up, the lentiform fork sign was further improved on brain MRI ( Figure 1d). However, at eight months after the onset, he still complained movement disorders, such as a wobble when walking and body tilting when resting. Brain lesions were still evident on CT scan ( Figure 1e).

## Discussion

Herein, we report a diabetic hemodialysis patient with consciousness disturbance who presented with the lentiform fork sign on T2-weighted brain MRI. This finding appears in the basal ganglia, which is vulnerable to addictive toxins and metabolic products ^8, 12^. The lentiform fork sign is comprised of the following elements: 1) the lateral arm, formed by the edematous external capsule and extending from the anterior end of the putamen to the stem; 2) the stem, created by merging of the edematous external and internal capsules at the inferoposterior end of the putamen; and 3) the medial arm, which extends from the stem anteriorly up to one third of the medial edge, where it splits into two slightly less T2/FLAIR-hyperintense branches engulfing the globus pallidus ^11, 12, 15^. In the present case, brain MRI showed the same expansile high signal intensity ( Figure 1b). The lentiform fork sign is rare but non-specific. Thus, a differential diagnosis should be considered ( Table 1) ^8, 11– 15^, of which ME or DUS may be the cause in the present case.

**Table 1.** Differential diagnosis of pathological conditions presenting with the lentiform fork sign ^8, 11– 15^.

a) Uremic encephalopathy *
b) Severe metabolic acidosis
c) Ketoacidosis
d) Dialysis disequilibrium syndrome
e) Intoxication (methanol, ethylene glycol, etc)
f) Drug-induced (metformin)

*The lentiform fork sign mainly occurs in patients with diabetic kidney disease.

The use of metformin in dialyzed patients can cause drug accumulation in the brain, leading to neurological abnormalities, difficulties of speech and walking, with worsening of sensory disturbance, tiredness, drowsiness, and weakness (i.e., ME) ^6, 7, 9, 10^. Metformin is first-line drug used in type 2 diabetes mellitus. However, it is contraindicated in patients with an estimated glomerular filtration rate <30 mL/min/1.73 m ^2^, because of an increased risk of lactic acidosis. Acidosis can damage the basal ganglia, resulting in cytotoxic edema ^7^, which is sometimes irreversible despite intensive hemodialysis to remove metformin and lactic acid, and to correct acidosis. According to the previous reports, hemodialysis patients are at risk for thiamin deficiency which is induced to encephalopathy, because they are in the condition of malnutrition and tend to lose water-soluble vitamins in the hemodialysis procedure ^16, 17^. Furthermore, thiamin deficiency may be a possible mechanism in metformin-induced encephalopathy ^18^. In our case, thiamin level was not decreased and severe lactic acidosis was not observed. In addition to these, the occurrence of gait disturbance, severe dysarthria, and consciousness disturbance was subacute even though sluggish speaking and dysarthria had been gradually worsening six months before starting taking metformin as shown in Figure 2, and the patient was neither malnutrition nor weight loss. That is why it was not necessarily ME.

Alternatively, DUS is characterized by acute or subacute progression with a variety of movement disorders such as gait disorders, dysarthria, parkinsonism, and consciousness disturbance. DUS can cause bilateral symmetrical basal ganglia lesions on brain CT and T2-weighted MRI ^4, 5^ in patients with diabetic nephropathy, even if they are not on hemodialysis. To date, approximately 30 cases of DUS have been reported, many of which are Asian. The reported risk factors of DUS include a high level of HbA1c before and at hemodialysis, and increasing metabolic acidosis. Hyperglycemia damages the microvasculature, resulting in a fragile vascular smooth muscle, and the accumulation of uremic toxins and/or metabolic acidosis can damage the blood-brain-barrier, leading to altered metabolism and homeostasis in the brain. This can result in basal ganglia injury, including angiogenic edema, which is reversible and shows favorable prognosis.

The clinical presentation in our case was not helpful for differentiating ME and DUS, because these symptoms were indistinguishable ( Table 2). Initial hemodialysis improved lactic acidosis, although intensive hemodialysis for six consecutive days was required to improve his consciousness. The lentiform fork sign on MRI improved at first, although brain CT findings at eight-month follow-up showed low density signals in those regions, and his neurological sequelae remained, suggestive of continued cytotoxic edema. ME was likely the main cause of injury in our case. Nevertheless, the patient’s condition worsened relatively rapidly before admission, similar to that seen in DUS. DUS can also contribute to cytotoxic edema in the basal ganglia, and has a variable progression. Thus, DUS may have also contributed to the encephalopathy in our case.

**Table 2.** Comparison with metformin-encephalopathy (ME) and diabetic uremic syndrome (DUS) ^4– 7, 11– 14^.

	ME	DUS	Present case
**Clinical findings**	sensorium, tiredness, dysarthria, gait disorder, consciousness disturbance	dysarthria, gait disorder, parkinsonism, consciousness disturbance	tiredness, dysarthria, gait disorder, consciousness disturbance
**Onset**	gradually and subacute	acute and subacute	gradually (dysarthria) and subacute (gait and consciousness disturbance)
**Acidosis**	Lactic acidosis	Metabolic acidosis	Lactic & metabolic acidosis
**Uremia**	-	+	-
**Hyperglycemia**	-	+	-
**CT**	low density area	low density area	low density area
**MRI (T2-weighted)**	high intense lesion	high intense lesion	high intense lesion
**Characteristic of edema**	cytotoxic	vasogenic	cytotoxic
**Therapy**	stop metformin intensive hemdialysis	intensive hemdialysis	intensive hemdialysis
**Prognosis**	remained	good	remained

ME, metformin-associated encephalopathy; DUS, diabetic uremic syndrome.

In summary, we report a diabetic hemodialysis patient with encephalopathy presenting as the lentiform fork sign derived from ME and/or DUS. In dialysis patients showing gait and consciousness disturbance, the lentiform fork sign on brain CT and T2-weighted MRI may be useful for differential diagnosis.

## Data availability

All data underlying the results are available as part of the article and no additional source data are required.

## Consent

Written informed consent for publication of their clinical details and clinical images was obtained from the patient.
