# Supplementary material for: Case Report: A Case of Encephalopathy Presenting the Lentiform Fork Sign on MRI in a Diabetic Dialysis Patient
Source: F1000Res. 2021 Dec 2;9:969. Originally published 2020 Aug 11. [Version 3] doi: 10.12688/f1000research.25597.3 (PMC8602957; doi:10.12688/f1000research.25597.3)
Supplement: Peer review history

# Peer review: Reviewer response for version 2

**DOI:** 10.5256/f1000research.78515.r97746

**Published:** 2021-11-18

**Authors:** Ping-Hsun Wu

**Affiliations:** 1 Division of Nephrology, Department of Internal Medicine, Kaohsiung Medical University Hospital, Kaohsiung Medical University, Kaohsiung, Taiwan

Yuri Ishizaki *et al.* reported a case of lentiform fork sign on MRI in a dialysis patient. The case presentation is good and well written. I have some questions about this case report.

1. The consciousness disturbance improved after intensive hemodialysis. The hemodialysis dosage and time interval could be mentioned in this paper.
2. Metformin was prescribed for diabetic hemodialysis patients. In this condition, metformin-associated lactic acidosis may present. In the revised main text, the author mentioned that the level of metabolic (lactic) acidosis on admission was not very high. Please provide the acidosis status (HCO3 level and lactate level) in the main text.

Are enough details provided of any physical examination and diagnostic tests, treatment given and outcomes?

Yes

Is the case presented with sufficient detail to be useful for other practitioners?

Yes

Is sufficient discussion included of the importance of the findings and their relevance to future understanding of disease processes, diagnosis or treatment?

Partly

Is the background of the case’s history and progression described in sufficient detail?

Yes

Reviewer Expertise:

Clinical nephrology, hemodialysis, chronic kidney disease

I confirm that I have read this submission and believe that I have an appropriate level of expertise to confirm that it is of an acceptable scientific standard.

# Response

**Published:** 2021-11-27

**Authors:** Yuri Ishizaki

**Affiliations:** University of Miyazaki, Japan

Dear Dr. Ping-Hsun Wu,

 Thank you for providing your valuable comments. Our responses are as follows.

**1) The consciousness disturbance improved after intensive hemodialysis. The hemodialysis** **dosage and time interval could be mentioned in this paper.**

 In the part of the Case report, we mentioned that the patient received intensive hemodialysis (four hours per day) for six days after hospitalization. We added the extra information about hemodialysis condition: quantity of blood flow of 200 mL/min, quantity of dialysate flow of 500 mL/min, 1.5 m ^2^ -dialysis membrane made of polymer alloy (polyarylate/polyethersulfone). After his consciousness recovered, we performed hemodialysis treatment regularly (four hours, three times a week).

**2) Metformin was prescribed for diabetic hemodialysis patients. In this condition, metformin-associated lactic acidosis may present. In the revised main text, the author mentioned that** **the level of metabolic (lactic) acidosis on admission was not very high. Please provide the** **acidosis status (HCO3 level and lactate level) in the main text.**

 As you mentioned, metformin-associated lactic acidosis may present. In our amendments from version 1 of the PDF file of this article, we considered and described that the level of metabolic(lactic) acidosis on admission was also much mild not so high. As written here, we modified “severe lactic acidosis was not observed” to “the level of metabolic acidosis on admission was not very high (bicarbonate ion, 18.1 mEq/L; lactic acid, 6.2 mmol/L)”.

 Sincerely yours,

 Yuri Ishizaki

# Peer review: Reviewer response for version 2

**DOI:** 10.5256/f1000research.78515.r97466

**Published:** 2021-11-08

**Authors:** Shashwati Sarkar Sen

**Affiliations:** 1 Department of Reproductive Medicine and Surgery, Mohak Superspeciality Hospital, Prayagraj, Uttar Pradesh, India

The queries and suggestions have been answered satisfactorily.

Are enough details provided of any physical examination and diagnostic tests, treatment given and outcomes?

Yes

Is the case presented with sufficient detail to be useful for other practitioners?

Yes

Is sufficient discussion included of the importance of the findings and their relevance to future understanding of disease processes, diagnosis or treatment?

Yes

Is the background of the case’s history and progression described in sufficient detail?

Yes

Reviewer Expertise:

Endocrine disorders, Assisted reproductive technology, Infertility, Gynaecology, Obstetrics

I confirm that I have read this submission and believe that I have an appropriate level of expertise to confirm that it is of an acceptable scientific standard.

# Response

**Published:** 2021-11-11

**Authors:** Yuri Ishizaki

**Affiliations:** University of Miyazaki, Japan

Dear Shashwati Sarkar Sen,

 Thank you for your valuable comments and suggestions.

 Sincerely,

 Yuri Ishizaki

# Peer review: Reviewer response for version 1

**DOI:** 10.5256/f1000research.28250.r95340

**Published:** 2021-09-27

**Authors:** Shashwati Sarkar Sen

**Affiliations:** 1 Department of Reproductive Medicine and Surgery, Mohak Superspeciality Hospital, Prayagraj, Uttar Pradesh, India

Summary: It is prudent to avoid Metformin in Diabetic patients with chronic renal insufficiency. Metformin toxicity can cause lactic acidosis induced encephalopathy eventually leading to multi-organ failure and death. Lentiform fork sign is a specific MRI finding in basal ganglia of these patients probably due to vasogenic edema induced by metabolic acidosis. Suspicion about Metformin intake will help in an early diagnosis and treatment.

 A few suggestions to the authors: 

1. Restrict the number of words in the title. Too many medical terms are used.
2. Case Report: Correction - Arterial Blood Gas analysis. 
3. Mention the Glasgow Coma Score.
4. DUS may have a subacute presentation. Yoshiko Nishimura *et al.* ^2^ **have reported a case of Diabetic Uremic Syndrome where the clinical features developed over 3 months. In your case, ME seems to be the primary diagnosis and DUS may be considered as a differential diagnosis rather than a contributory factor.

Add these References:  

1. Metformin-induced encephalopathy: the role of thiamine ^1^.McGarvey *et al. *(2018) suggested that Metformin may cause thiamine deficiency in patients with end-stage diabetic renal failure, resulting in a specific type of encephalopathy. A quick resolution of the signs and symptoms was observed on stopping the drug in these patients.
2. A case of subacute Parkinsonism presenting as bilateral basal ganglia lesions by MRI in Diabetic uremic Syndrome. ^2^

Are enough details provided of any physical examination and diagnostic tests, treatment given and outcomes?

Yes

Is the case presented with sufficient detail to be useful for other practitioners?

Yes

Is sufficient discussion included of the importance of the findings and their relevance to future understanding of disease processes, diagnosis or treatment?

Yes

Is the background of the case’s history and progression described in sufficient detail?

Yes

Reviewer Expertise:

Endocrine disorders, Assisted reproductive technology, Infertility, Gynaecology, Obstetrics

I confirm that I have read this submission and believe that I have an appropriate level of expertise to confirm that it is of an acceptable scientific standard.

## References

[1] : Metformin-induced encephalopathy: the role of thiamine. *Intern Med J* .48(2) : 10.1111/imj.13693 194-197 10.1111/imj.13693 29415360

[2] : [A case of subacute parkinsonism presenting as bilateral basal ganglia legions by MRI in diabetic uremic syndrome]. *Rinsho Shinkeigaku* .2013;53(3) : 10.5692/clinicalneurol.53.217 217-23 10.5692/clinicalneurol.53.217 23524602

# Response

**Published:** 2021-10-16

**Authors:** Yuri Ishizaki

**Affiliations:** University of Miyazaki, Japan

Dear Dr. Shashwati Sarkar Sen,

 Thank you for your valuable comments about my case report. We respond to each suggestion as follows.

**1) Restrict the number of words in the title. Too many medical terms are used.**

 As suggested by the reviewer, we shorten the title to “A Case of Encephalopathy Presenting the Lentiform Fork Sign on MRI in a Diabetic Dialysis Patient”.

**2) Case Report: Correction - Arterial Blood Gas Analysis.**

 Thank you for pointing it out.  We corrected “Atrial blood gas” to “Arterial blood gas analysis”. 

**3) Mention the Glasgow Coma Score.**

  We mentioned the Glasgow Coma Scale Score of E3V4M6 in the first line of the case report section.

**4) DUS may have a subacute presentation. Yoshiko Nishimura *et al. *have reported a case of Diabetic Uremic Syndrome where the clinical features developed over 3 months. In your case, ME seems to be the primary diagnosis and DUS may be considered as a differential diagnosis rather than a contributory factor.**

 Thank you for your comment. In our case, there was not enough evidence to diagnose ME. The first reason was the thiamin level was not decreased, even though metformin may induce thiamin deficiency. (We added serum thiamin value in the part of the case presentation.) Furthermore, thiamin deficiency can be caused by malnutrition and loss of water-soluble vitamins in the hemodialysis progression, but the patient did not have the symptoms of weight loss and seemed not to be undernourished. Secondly, the level of metabolic (lactic) acidosis on admission was also much mild not so high. Thirdly, as shown in Fig. 2, although the patient had had sluggish speaking and dysarthria and these findings had been gradually worsening six months before starting taking metformin, the relatively rapid-onset gait disturbance, severe dysarthria, and consciousness disturbance had appeared ten days before admission, which was a subacute progression. We could say that it was difficult to distinguish between ME and DUS, just considering the appearance of the findings. That is why we cannot necessarily diagnose ME, and we listed the two differential diagnoses, ME and/or DUS.

 We added the following sentences in the Discussion section:

 According to the previous reports, hemodialysis patients are at risk for thiamin deficiency which is induced to encephalopathy, because they are in the condition of malnutrition and tend to lose water-soluble vitamins in the hemodialysis procedure [16, 17]. Furthermore, thiamin deficiency may be a possible mechanism in metformin-induced encephalopathy [18]. In our case, the thiamin level was not decreased and severe lactic acidosis was not observed. In addition to these, the occurrence of gait disturbance, severe dysarthria, and consciousness disturbance was subacute even though sluggish speaking and dysarthria had been gradually worsening six months before starting taking metformin as shown in Fig.2, and the patient was neither malnutrition nor weight loss. That is why it was not necessarily ME.

**References**

 We also read the two references which you indicated. According to Reference #1, thiamine must be a key to diagnose Metformin-induced encephalopathy. In the part of that we showed the thiamin level of our case, we quoted reference #1. On the other hand, we did not add reference #2 because it is written in Japanese and we thought that reader cannot understand it well.

 Furthermore, we quoted two more references to mention that our case is not necessarily ME in the perspective of thiamin level in hemodialysis patients (Szu-Chun H et al, AJKD, 2001).

 Sincerely yours,

 Yuri Ishizaki
